# Exploring the clinical value of quantitative ultrasound scoring method in optimizing the classification of breast BI-RADS category 4a nodules in the Tibetan Plateau region

**DOI:** 10.3389/fonc.2026.1643787

**Published:** 2026-05-13

**Authors:** Min Sun, Daozu Yuan, Hongmei Liu, Xiaoqing Wang, Xia Wu, Rong Xiao

**Affiliations:** 1 Department of Ultrasound Medicine, Hospital of Chengdu Office of People’s Government of Tibetan Autonomous Region (Hospital.C.T.), Chengdu, China; 2 Department of Cancer Center, The Seventh People’s Hospital of Chengdu, Chengdu, China

**Keywords:** breast, category 4a nodules, optimal classification, plateau region, quantitative scoring method

## Abstract

**Objective:**

This study aimed to evaluate the clinical utility of a quantitative ultrasound (US) scoring method in refining the classification of Breast Imaging Reporting and Data System (BI-RADS) Class 4a breast nodules in patients from the Tibetan Plateau region.

**Methods:**

A retrospective analysis was conducted on the US and contrast-enhanced US (CEUS) imaging characteristics of 275 breast nodules from high-altitude regions. Significant US features were identified and assigned scores using chi-square tests and binary logistic regression analysis. Separate scores for each nodule were calculated based on US and CEUS findings. Receiver operating characteristic curves were generated to determine the optimal cutoff value, which served as the basis for classification refinement. Nodules with scores below the cutoff value were reclassified as Category 3, while those with one or more scores meeting or exceeding the cutoff value remained in Category 4a. The differences in biopsy rates and malignant detection rates for Category 4a nodules were assessed before and after applying the optimized classification system.

**Results:**

All 275 nodules were pathologically confirmed, with 148 classified as benign and 127 as malignant. Regression analysis of US features revealed significant differences between benign and malignant nodules regarding the boundary, morphology, internal echo, resistance index, and hyperechoic halo (*P < 0.05*). Post-regression analysis of CEUS features indicated significant differences between benign and malignant lesions in enhancement intensity, enhancement boundary, perfusion defects, range of enhancement, and the presence of a crab-foot sign (*P < 0.05*). The optimal cutoff value for both US and CEUS scoring was determined to be 7.5 points. Optimized classification was applied to 97 BI-RADS 4a nodules, resulting in 67 nodules being downgraded to Category 3, while 30 remained in Category 4a. Following optimized classification, the biopsy rate decreased to 30.93%, with a significant difference in both biopsy and malignant detection rates before and after optimized classification (*P < 0.05*).

**Conclusion:**

Quantitative US scoring serves as an effective index for optimizing the classification of BI-RADS Category 4a breast nodules in high-altitude regions. This method enhances diagnostic accuracy, significantly reduces unnecessary biopsy procedures, and provides a reliable framework for precise clinical diagnosis and treatment planning.

## 1 Introduction

Breast cancer is the most prevalent malignant tumor among women globally, posing a severe threat to their physical and mental health. Early diagnosis and treatment are critical for reducing breast cancer mortality and improving patients’ quality of life. The occurrence and development of breast cancer have significant geographical variability (1, 2). In high-altitude regions, low temperatures and hypoxic conditions are known to increase the risk of cancer development (3–5), contributing to differences in gene expression and imaging characteristics (1, 6).

The Breast Imaging Reporting and Data System (BI-RADS) was first proposed by the American Society of Radiology in 1992 and has been revised several times and introduced in a fifth edition in 2013 (7).The BI-RADS, a system for characterizing breast lesions, assessing the lesion categories, and giving recommendations and auditing parameters to facilitate standardized diagnosis and management of breast disease, is now widely used in medical institutions at all levels worldwide (8–11).Although the BI-RADS system provides a standardized framework for the ultrasonographic diagnosis of breast diseases, the complexity of breast pathology often results in overlapping lesion presentations. BI-RADS 4a lesions carry an estimated 2%–10% risk of malignancy (7, 12). Guidelines recommend using needle biopsy, leading to over 90% of benign lesions undergoing invasive procedures, thereby resulting in overtreatment and resource wastage. This contradiction is particularly pronounced in high-altitude regions with limited medical resources, where biopsy implementation is challenging, complications are high, patient follow-up compliance is poor, and misdiagnosis rates are elevated. How to reduce biopsies of benign lesions while minimizing missed diagnoses of malignancies has become an urgent clinical priority.

The current BI-RADS classification relies on gray-scale ultrasound morphological features, with strong subjectivity in defining category 4a and poor observer agreement. Contrast-enhanced ultrasound (CEUS) reflects microcirculatory perfusion; however, in clinical practice, interpretation often relies on visual qualitative assessment without objective quantitative thresholds. Its diagnostic value in low-suspicion lesions remains controversial. These limitations frequently lead to overutilization of biopsy for benign 4a nodules and underdiagnosis of atypical malignant nodules.

Recently, advanced imaging/CAD methods have evolved rapidly, demonstrating substantial potential to improve diagnostic accuracy, reduce interpretation variability, and facilitate personalized treatment approaches (13, 14). However, their limitations have become increasingly apparent. Most imaging/CAD systems rely on high-end imaging equipment and extensive datasets, presenting high operational barriers that make them difficult to adapt for resource-constrained high-altitude regions. Concurrently, some studies on advanced imaging/CAD methods remain confined to qualitative assessments or complex quantitative modelling, lacking unified, straightforward evaluation tools and thus encountering difficulties in clinical translation. Consequently, clinical decision-making for BI-RADS 4a lesions remains clinically challenging. The imperative to minimise unnecessary biopsies, alleviate patient burden, and reduce health care resource waste, particularly in resource-constrained high-altitude regions, while ensuring no malignant lesions are missed, is of paramount importance.

Due to the scarcity of medical resources in high-altitude regions, it is challenging to implement advanced imaging/CAD methods. This study developed a quantitative model based on US and CEUS imaging features to achieve precise and convenient stratification of BI-RADS 4a nodules. Key advantages include a straightforward and highly implementable approach that can be directly integrated into routine clinical practice, making it suitable for high-altitude regions with limited medical infrastructure. Second, standardised quantitative criteria reduce reliance on operator experience, enhancing objectivity and consistency of results. Finally, it provides reliable quantitative evidence for clinical practice, improving the detection rate of malignant lesions while reducing the risk of unnecessary needle biopsies for benign lesions. This study fills a gap in the precise stratification assessment of BI-RADS 4a nodules in medically underserved regions, effectively bridging the gap in the clinical translation of advanced technologies. It demonstrates clear clinical application value and promising prospects for widespread adoption.

This study employs a quantitative model constructed from ultrasound and contrast-enhanced ultrasound (CEUS) core features, which effectively distinguishes benign from malignant BI-RADS 4a lesions. It is hypothesised that its diagnostic performance (sensitivity, specificity, and AUC) is superior to conventional qualitative methods, enabling precise identification of malignant lesions requiring biopsy and benign lesions suitable for follow-up observation. This approach effectively resolves the clinical decision-making challenges associated with BI-RADS 4a nodules in high-altitude regions.

## 2 Methods

### 2.1 Subject of the study

This study included 274 female patients who underwent routine breast US and contrast-enhanced ultrasound (CEUS) between April 2022 and May 2024 at the Hospital of the People’s Government of the Tibet Autonomous Region in Chengdu. If multiple nodules are present within a single breast, the lesion with the highest BI-RADS classification is selected. If two or more nodules share the same BI-RADS category, the largest in diameter is chosen. If nodules in both breasts independently meet the inclusion criteria, they may be enrolled simultaneously. A total of 275 breast nodules were analyzed, including 97 categorized as BI-RADS 4a. The patients ranged in age from 13 to 83 years, with a mean age of 48.0 ± 12.1 years. Nodule diameters ranged from 0.5 to 14.3 cm, with a mean diameter of 2.2 ± 1.1 cm.

**Figure 1:**
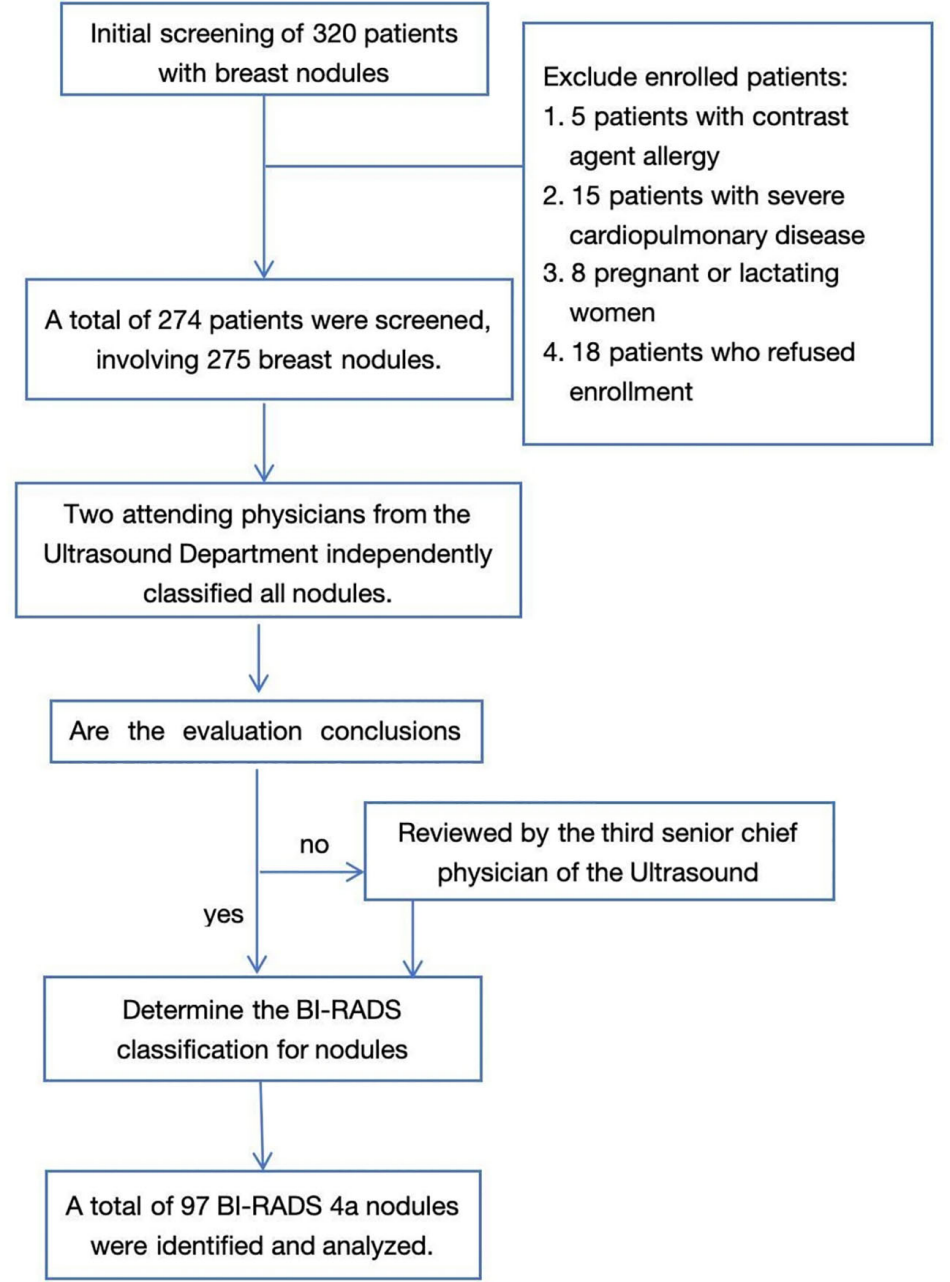
Incorporate into the screening flowchart.

The inclusion criteria were as follows: Patients residing at altitudes of ≥ 2000 m for at least five years, lesions evaluated using both US and CEUS before surgery, and lesions with confirmed pathological findings. The exclusion criteria were as follows: including patients with contrast agent allergies, severe cardiopulmonary insufficiency (such as heart failure or respiratory failure), acute coronary syndrome, severe pulmonary hypertension (pulmonary artery pressure > 90 mmHg), and women who are pregnant or lactating. Our clinical workflow for screening patient contraindications included the following steps. Step One: Systematic Pre-screening. We systematically reviewed previous imaging reports and diagnostic records via the electronic medical records system to exclude patients with a documented allergy to sulfur hexafluoride contrast agents, New York Heart Association (NYHA) Class III/IV heart failure, long-term home oxygen therapy, or those who were pregnant or breastfeeding. Step Two: Structured Interview and Questionnaire. A structured interview and questionnaire were administered by trained research nurses on the day of enrollment. Pulmonary hypertension was defined based on right heart catheterization or echocardiography measurements within the preceding 3 months, with a documented systolic pulmonary artery pressure ≥ 90 mmHg (15) Respiratory failure was defined as persistent resting oxygen saturation < 90% or an arterial partial pressure of oxygen < 60 mmHg. Acute coronary syndrome was defined as symptom onset ≤ 30 days before enrollment, confirmed by electrocardiographic findings and troponin testing. For women of childbearing potential, the date of the last menstrual period and lactation history were recorded. Step Three: Review and Confirmation. All positive or questionable medical histories were reviewed by a senior internist, with reference to the original medical records when necessary. Patients meeting any exclusion criteria were not enrolled, with detailed documentation of reasons for exclusion. (**Figure 1**).The study protocol was designed according to the Declaration of Helsinki and approved by the Ethics Committee on Biomedical Research at the Chengdu Office Hospital of the People’s Government of Tibet Autonomous Region. Written informed consent was waived.

### 2.2 Instrumentation and ultrasonography

#### 2.2.1 Equipment and probes

During the study period from April 2022 to May 2024, routine ultrasound examinations were conducted using the Mindray Resona R9, 7, or the French Sanofi Supersoni Imaging Aixplorer colour Doppler ultrasound diagnostic systems. These employed L12–5, L15–3, or L15–4 transducers with frequencies of 5–12, 3–15, and 4–15 MHz, respectively. Contrast-enhanced ultrasound was performed using the Mindray Resona R9 and 7 systems with L9–3 transducers (frequency range: 3–9 MHz), maintaining a mechanical index between 0.06 and 0.08. All three devices represent standard clinical configurations. The contrast-enhanced imaging systems are manufactured by the same manufacturer, with imaging modes calibrated by the manufacturer for consistency. Equipment allocation was randomized according to the patient-enrollment sequence. To mitigate systematic device-to-device variation, all ultrasound images and videos were stored for subsequent analysis.

#### 2.2.2 Standardized image acquisition process

Images were acquired by two physicians with over 10 years of experience in conventional ultrasound and at least 3 years of experience in contrast-enhanced ultrasound. Before the examinations, both physicians underwent standardized training and passed a consistency evaluation. The entire imaging process strictly adhered to a standardized protocol (16, 17), thereby minimizing the impact of operator subjectivity.

On conventional ultrasound, The patient was positioned supine or in the lateral decubitus position, with both breasts and axillae fully exposed. The imaging depth was adjusted to include the lesion and surrounding normal glandular tissue (typically 3–5 cm). A single focal zone was placed at the lower margin of the lesion, and the gain was adjusted to achieve a medium echo pattern in normal glandular tissue, while maintaining a dynamic range of 60–70 dB. Routine transverse and longitudinal scans of the lesion were performed, documenting its location, size (measured by the longest diameter), morphology, margins, internal echotexture, calcifications, and flow signals. For each lesion, at least two static images, each in transverse and longitudinal planes, were stored, along with a measurement diagram including a scale.

During ultrasound contrast imaging, the two-dimensional maximum cross-sectional view of the lesion that appeared suspicious or demonstrated abundant blood flow signals was selected. The lesion was positioned at the centre of the image, with adequate normal glandular tissue visible on both sides. For contrast administration, dual-window contrast mode was selected, using sulfur hexafluoride microbubbles (SonoVue, Bracco). Before use, 5 mL saline solution was injected, and the vial was shaken thoroughly to ensure complete mixing. A 4.8 mL per bolus was administered rapidly via the antecubital vein, followed by a 5–10 mL saline flush. Low-mechanical index contrast mode (mechanical index 0.06–0.08) was initiated, with the gain adjusted to eliminate background noise. The imaging depth and focal position were maintained consistently with grey-scale ultrasound settings, and the frame rate was set at 10–15 Hz. Timing was initiated immediately upon injection of the contrast agent, and continuous dynamic observation was performed for ≥ 3 min, with the full dynamic DICOM sequence stored.

In this study, the maximum lesion diameter was measured independently by two physicians without knowledge of the pathological results. The largest cross-sectional plane demonstrating suspicious features on two-dimensional ultrasound or abundant blood flow signals was selected, while avoiding regions containing a high proportion of anechoic areas or coarse calcifications within the lesion. Each lesion was measured three times, and the mean value was recorded. If the difference among the three measurements exceeded 10%, the imaging plane was re-scanned, and measurements were repeated.

Additionally, the intraclass correlation coefficient (ICC) was used to evaluate inter-operator consistency of results. Two senior ultrasound consultants independently measured 20 randomly selected BI-RADS 4a lesions. The calculated intraclass correlation coefficient (ICC) was 0.91 (95% CI: 0.85–0.93). This result indicates excellent inter-operator measurement consistency, thereby effectively ensuring the reliability of measurement outcomes and mitigating confounding effects arising from operator-dependent variability. The patient was positioned supine, with full exposure of the bilateral breasts and axilla. During the US examination, images of different lesion sections were stored, and information such as nodule size, position, and blood flow signals were recorded. For CEUS, the section with the largest lesion diameter was selected as the optimal display view, ensuring simultaneous visualization of the lesion and surrounding normal tissues. A dual-amplitude control mode was employed, in which 4.8 mL of the prepared contrast suspension was rapidly administered via the elbow vein, followed by a flush with 5–10 mL of physiological saline. Dynamic video recordings of the imaging, lasting no less than three minutes, were stored for analysis.

### 2.3 Image analysis and assignment methods

This study is based on a systematic analysis of the strength of association between ultrasound and contrast-enhanced ultrasound features and the risk of breast malignancy across a substantial body of literature. It strictly adheres to previously reported scoring criteria (18–20) and incorporates extensive consultations with three ultrasound specialists possessing decades of extensive clinical experience. A rigorously predefined and standardized scoring system was adopted. Second, to validate the rationality of the preset scores in this scoring system, we first conducted univariate analyses of all ultrasound and contrast-enhanced ultrasound features to identify those associated with benign and malignant breast nodules, and performed multicollinearity tests (excluding features with VIF > 5). Next, the selected features were included in the logistic regression models of ultrasound and contrast-enhanced ultrasound, and variables that did not make a statistically independent contribution in the regression analysis were excluded, with only ultrasound and contrast-enhanced ultrasound features independently associated with malignant risk retained. Third, the predefined scores corresponding to the retained features were summed to calculate the total score for each nodule, thereby achieving the integration and quantification of the discriminatory performance of multiple features. The US evaluation and scoring criteria were based on the following parameters. For nodule boundary, scores ranged from 1 to 4 points: clear (1 point), mostly clear (2 points), less clear (3 points), and unclear (4 points). Nodule morphology was scored as regular (1 point), slightly irregular (2 points), and irregular (3 points). The presence of a burr sign was scored as absent (0 points) or present (1 point). Calcification was scored as absent (0 points), calcification diameter ≥ 0.5 mm (1 point), and microcalcification (2 points). The aspect ratio was scored as < 0.8 (0 points), 0.8 ≤ aspect ratio < 1 (1 point), and ≥ 1 (2 points). Internal echo was scored as hyperechoic or isoechoic (1 point), hypoechoic (2 points), and very low echo or glandular structural disorder (3 points). Internal echo uniformity was scored as uniform (0 point), slightly uneven (1points), and uneven (2points). Posterior echoes were scored as enhancement (0 points), unchanged (1 point), and attenuation (2 points). Blood flow signals were scored as no blood flow (0 points), grade I blood flow (1 point), grade II blood flow (2 points), and grade III–IV blood flow (3 points). Abnormal axillary lymph nodes were scored as absent (0point) or present (1points). The resistance index (RI) was scored as < 0.7 or absent (0 point) and ≥ 0.7 (1 points). The presence of a hypoechoic halo was scored as absent (0 points) or present (1 point).

The CEUS evaluation and scoring criteria were defined as follows. For enhancement speed, scores were assigned as slow progression (1 point), same progression (2 points), and fast progression (3 points). Enhancement intensity was scored as unenhanced (0 points), low enhancement (1 point), equal enhancement (2 points), and high enhancement (3 points). The enhancement boundary was scored as clear (1 point), mostly clear (2 points), and unclear (3 points). Enhancement morphology was scored as regular (1 point), irregular (2 points), and difficult to distinguish (3 points). Perfusion defects were scored as absent (0 points) or present (1 point). Enhancement homogeneity was scored as uniform (1 point) or non-uniform (2 points). The range of enhancement was scored as narrowing (1 point), unchanged (2 points), or expanding (3 points). The presence of a crab-foot sign was scored as absent (0 points) or present (1 point). Each criterion allowed only one score to be selected per item.

### 2.4 BI-RADS 4a nodule optimization classification criteria

The optimized classification of BI-RADS 4a nodules was determined based on the cutoff values from the two examination methods. Nodules with scores below the cutoff values for both methods were downgraded to Category 3, with follow-up recommended. Nodules with scores meeting or exceeding the cutoff values for either method remained in Category 4a, for which puncture biopsy was advised.

### 2.5 Statistical methods

Statistical analysis was performed using Statistical Package for the Social Sciences software (version 27.0). Measurement data are expressed as x ± s, while count data are presented as frequencies or percentages. Regarding the variable selection process, before constructing the multivariate logistic regression model, we first conducted a chi-square test was applied to analyze the conventional US and CEUS features of breast nodules through one-way analysis to identify statistically significant indicators. To mitigate the impact of multicollinearity on model stability, all selected variables underwent collinearity assessment using the variance inflation factor (VIF) as the criterion. Variables with VIF > 5 were excluded to ensure no severe multicollinearity was present among independent variables. Upon examination, the VIF values for all variables in this study were found to be < 5 (maximum VIF = 3.577).We then applied the Benjamini-Hochberg correction to control the false discovery rate (FDR) at the 0.05 level. Multivariate analysis was performed using backward stepwise regression for variable selection. This method incorporates all variables identified in univariate analysis into the model and progressively eliminates those with the highest P-values until all remaining variables achieve statistical significance (P < 0.05). This process ultimately identifies ultrasound features independently associated with the risk of malignancy in breast nodules at high altitudes, enabling the construction of a scoring model based on these findings. The scores of the variables included in the regression model were summed, and the receiver operating characteristic (ROC) curve was plotted using GraphPad Prism. The area under the curve (AUC) and the corresponding cutoff value were calculated. Statistical significance was set at P < 0.05.

## 3 Results

### 3.1 General information

This study included 274 patients with 275 breast nodules. Pathological diagnosis, used as the gold standard, identified 148 benign nodules (53.82%) and 127 malignant nodules (46.18%). Among the 97 BI-RADS Category 4a nodules screened for optimal classification, 9 (9.28%) were malignant. These included 7 cases of invasive ductal carcinoma, 1 case of ductal carcinoma *in situ*, and 1 case of mucinous carcinoma. The remaining 88 nodules (90.72%) were benign, comprising 35 fibroadenomas (including 1 with mucinous change), 18 adenopathies 23 intraductal papillomas, 7 cases of fibrocystic disorder, 2 cases of scarring, and 1 case each of sclerosing adenopathy, hamartoma, and lymphocytic mastitis. as depicted in **Table 1**.

**Table 1:** Comparison of demographic in 97 patients with BI-RADS 4a nodules.

Baseline characteristics	Benign (88)	Malignant(9)
Age(year)	43.63 ± 10.49	44.89 ± 7.54
Gender, male/female(n)	0/88	0/9
Tibetan(n, %)	82(93.18%)	9(100%)
Pathology		
Ductal carcinoma		7
Ductal carcinoma in situ		1
Mucinous carcinoma		1
Fibroadenomas	35	
Adenopathies	18	
Intraductal papillomas	23	
Fibrocystic disorder	7	
Scarring	2	
Sclerosing adenopathy	1	
hamartoma,	1	
Lymphocytic mastitis	1	

Age are presented as mean ± SD for continuous variables. Gender, Tibetan and Pathology are the number of lesions with percentages in parentheses.

### 3.2 Ultrasound characterization of benign and malignant breast nodules

**Figure 2:**
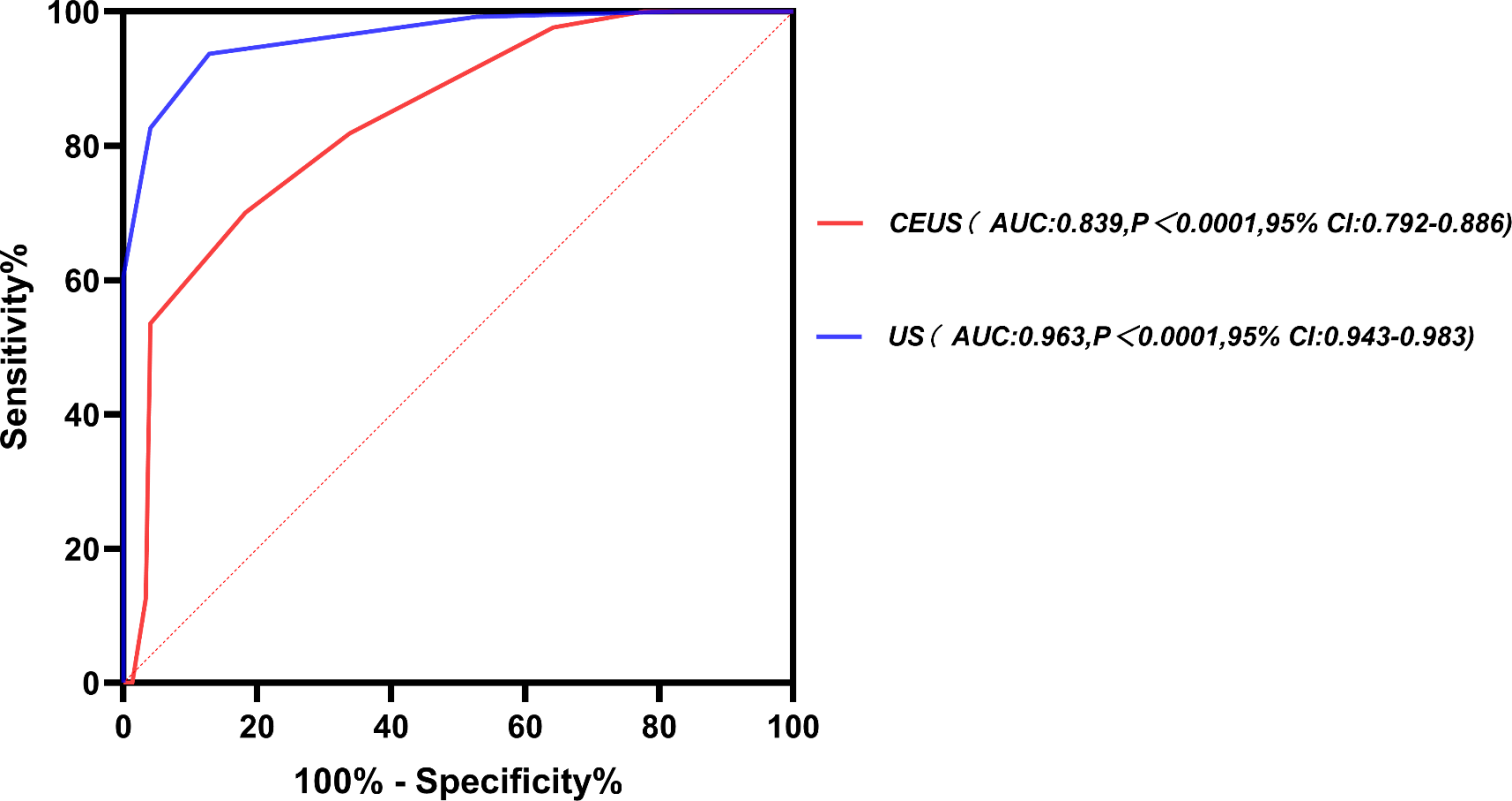
ROC curves for the diagnosis of malignant breast nodules using conventional US and CEUS scoring methods.

Univariate analysis revealed significant differences in several US features between benign and malignant breast lesions (P < 0.05). These features included boundary, morphology, burr sign, calcification, internal echo, internal echo uniformity, posterior echoes, blood flow signals, abnormal axillary lymph nodes, RI, and hyperechoic halo, as depicted in **Table 2**. Similarly, CEUS features such as enhancement during the solid phase, enhancement intensity, post-enhancement border, post-enhancement morphology, perfusion defects, enhancement uniformity, post-enhancement range, and the presence of a crab-foot sign were also significantly different between benign and malignant lesions (P < 0.05), as indicated in **Table 3**. The significant features from both US and CEUS analyses were incorporated into their respective regression models, with results presented in **Tables 4**, **5**. After regression analysis, the scores of US signs that remained statistically significant were summed. The AUC values for conventional US and CEUS were 0.962 and 0.834, respectively (**Figure 2**). The optimal cutoff value for both modalities was determined to be 7.5. For conventional US scores, the sensitivity, specificity, accuracy, and 95% confidence intervals were 0.937, 0.872, 0.896, and 0.943-0.983,respectively. For CEUS scores, the sensitivity, specificity, accuracy, and 95% confidence intervals were 0.701, 0.818, 0.808,and 0.792-0.886, respectively.

**Table 2:** Results of one-way analysis of routine US characteristics of breast nodules.

US performance		Benign (n = 148)	Malignant (n = 127)	X²	P
Nodule boundary	Clear	79	6	121.824	0.000^*^
	Clearer	28	6		
	Less clear	32	51		
	Unclear	9	64		
Nodule morphology	Regular	40	4	97.968	0.000^*^
	Less than regular	88	33		
	Irregular	20	90		
Burr sign	No	147	63	93.604	0.000
	Yes	1	64		
Calcification	No	133	50	84.055	0.000^*^
	Calcification diameter ≥ 0.5 mm	8	17		
	Microcalcification	7	60		
Aspect ratio	< 0.8	78	69	0.540	0.763
	0.8 ≤ aspect ratio < 1	40	30		
	> 1	30	28		
Internal echo	Hyperechoic or isoechoic	15	9	118.869	0.000^*^
	hyperechoic	107	14		
	Very low echo or glandular structural disorders	26	104		
Internal echo uniformity	Uniform	115	37	67.860	0.000^*^
	slightly uneven	9	41		
	Uneven	24	49		
Posterior echoes	enhancement	15	21	13.332	0.010^*^
	Unchanged	85	45		
	attenuation	48	61		
Blood flow signals	No blood flow	63	14	57.786	0.000^*^
	I blood flow	59	40		
	II blood flow	18	51		
	III–IV blood flow	8	22		
Abnormal axillary lymph nodes	No	148	51	122.391	0.000^*^
	Yes	0	76		
RI	< 0.7 or none	142	51	101.652	0.000^*^
	≥ 0.7	6	76		
Hypoechoic halo	No	145	86	44.303	0.000^*^
	Yes	3	41		

RI, Resistance Index.

P-values were calculated using the Chi-square test (or Fisher’s exact test when expected frequency < 5) if indicated.

*Indicates P < 0.05 after Benjamini-Hochberg correction.

**Table 3:** Results of one-way analysis of CEUS characteristics of breast nodules.

Enhancement methods		Benign (n = 148)	Malignant (n = 127)	X²	P
Enhancement speed	Slow progress	49	13	33.015	0.000^*^
	Same progress	40	21		
	Fast progress	59	93		
Enhancement intensity	Unenhanced	13	0	40.490	0.000^*^
	Low enhancement	41	9		
	Equal enhancement	31	22		
	High enhancement	63	96		
Enhancement boundary	Clear	100	29	82.320	0.000^*^
	Less clear	17	80		
	unclear	31	18		
Enhancement morphology	Regular	94	37	58.094	0.000^*^
	Irregular	21	72		
	Difficult to distinguish	33	18		
Perfusion defects	No	128	83	17.093	0.000^*^
	Yes	20	44		
Enhancement homogeneity	Uniform	107	70	8.794	0.003^*^
	Non-uniformity	41	57		
Range of enhancement	Narrowing	18	10	66.606	0.000^*^
	Unchanged	91	22		
	Expanding	39	95		
Crabs foot sign	None	124	58	44.363	0.000^*^
	Some	24	69		

P-values were calculated using the Chi-square test (or Fisher’s exact test when expected frequency < 5) if indicated.

*Indicates P < 0.05 after Benjamini-Hochberg correction.

**Table 4:** Logistic regression analysis of routine US characteristics of breast nodules.

US performance	B	Wald	P	Exp (B)	95% CI
Nodule boundary	1.207	7.434	0.006	3.344	1.404–7.962
Nodule morphology	1.192	4.781	0.029	6.799	1.220–37.898
Burr sign	2.848	1.062	0.303	17.253	0.077–3887.036
Calcification	0.963	2.780	0.095	2.619	0.845–8.1214
Internal echo	3.363	17.249	0.000	37.718	6.800–209.199
Internal echo uniformity	0.025	0.002	0.962	1.025	0.375–2.798
Posterior echoes	–0.262	0.233	0.629	0.770	0.266–2.226
Blood flow signals	–0.150	0.113	0.736	0.861	0.361–2.056
Abnormal axillary lymph nodes	25.618	0.000	0.993	133.756	0.000–
RI	4.078	10.134	0.001	59.045	4.794–727.238
Hypoechoic halo	4.147	5.900	0.015	63.249	2.227–1796.247
Constant	–19.060	20.775	0.000	0.000	

CI, confidence interval; Exp (B), Exponentiated B.

P-values were calculated using the backward stepwise regression if indicated.

**Table 5:** Results of logistic regression analysis of CEUS characteristics of breast nodes.

Enhancement methods	B	Wald	P	Exp (B)	95% CI
Enhancement speed	0.305	1.903	0.168	1.357	0.879–2.094
Enhancement intensity	0.697	9.999	0.002	2.008	1.303–3.092
Enhancement boundary	1.213	9.394	0.002	3.362	1.548–7.300
Enhancement morphology	–0.551	2.056	0.152	0.576	0.271–1.224
Perfusion defects	1.334	8.055	0.005	3.797	1.511–9.54
Enhancement homogeneity	0.237	0.352	0.553	1.268	0.579–2.777
Range of enhancement	0.712	7.644	0.006	2.038	1.230–3.376
Crabs foot sign	1.144	10.779	0.001	3.140	1.586–6.217
Constant	–6.400	39.653	0.000	0.002	

CI, confidence interval; Exp (B), Exponentiated B.

P-values were calculated using the backward stepwise regression if indicated.

### 3.3 Results after optimized classification of class 4a nodules

**Figure 3:**
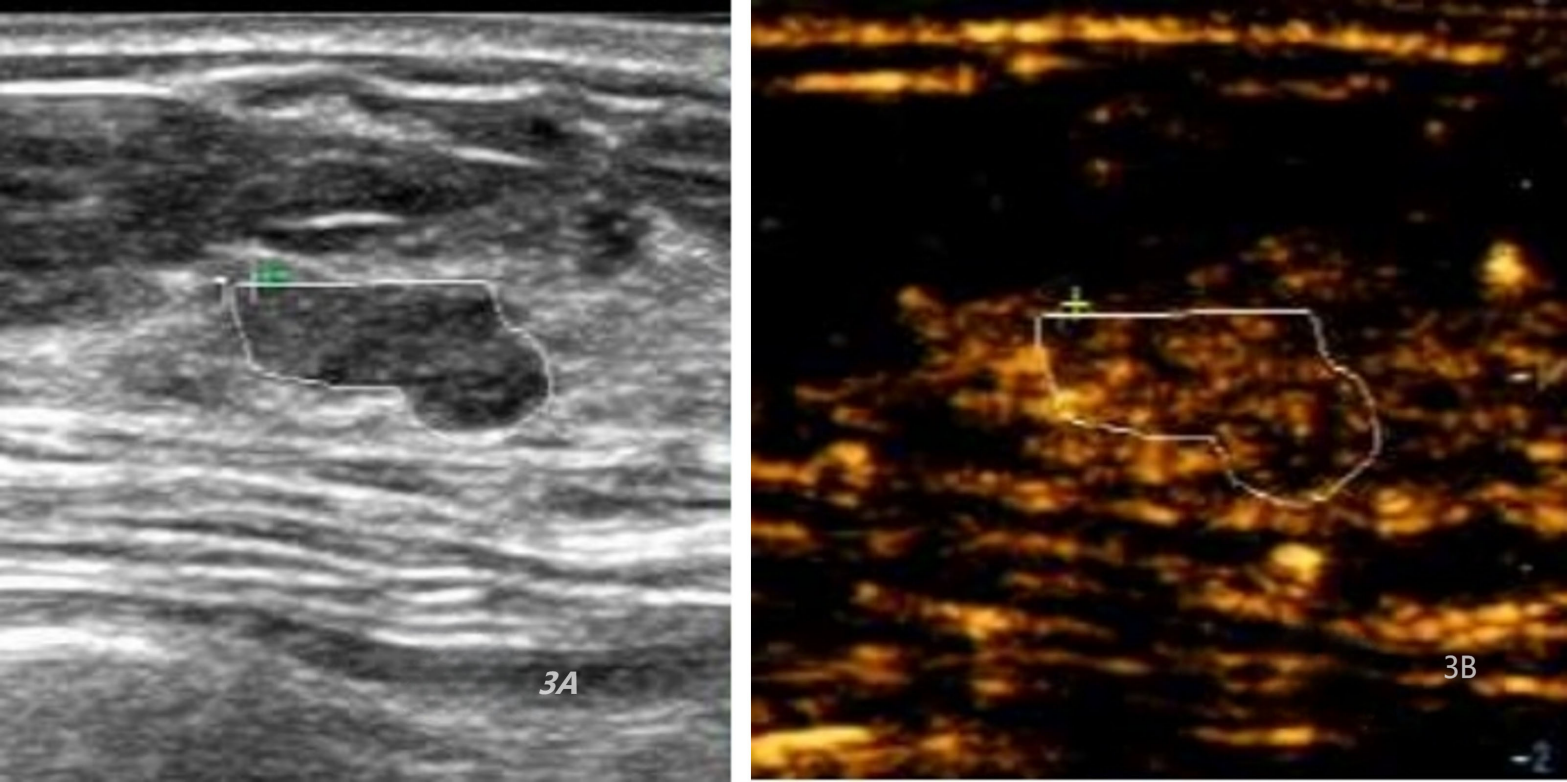
Ultrasound and CEUS images of a 40-year-old female patient with adenosis. **(A)** Ultrasound (US) image indicating a hypoechoic lesion with clear boundaries and irregular morphology, resulting in a total score of 6. **(B)** Contrast-enhanced ultrasound (CEUS) image depicting homogeneous equal enhancement, indistinct enhancement borders,resulting in a total score of 6.

**Figure 4:**
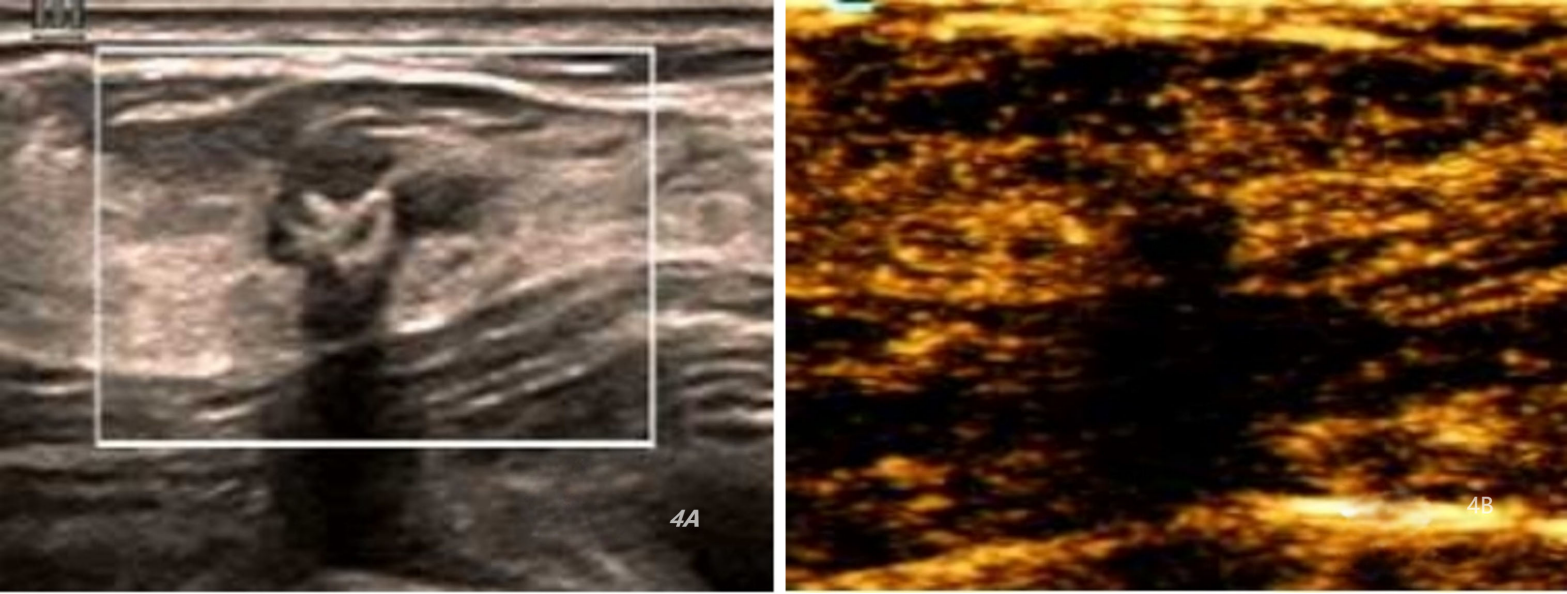
Ultrasound and CEUS images of a 32-year-old female patient with scleroing adenosis with calcifications. **(A)** Ultrasound (US) image indicating a hypoechoic lesion with mostly clear borders and slightly irregular morphology, and macrocalcifications within the area of adenosis. resulting in a total score of 6. **(B)** Contrast-enhanced ultrasound (CEUS) image depicting homogeneous equal enhancement, indistinct enhancement borders, and the lesion develops a focal perfusion defect. resulting in a total score of 7.

**Figure 5:**
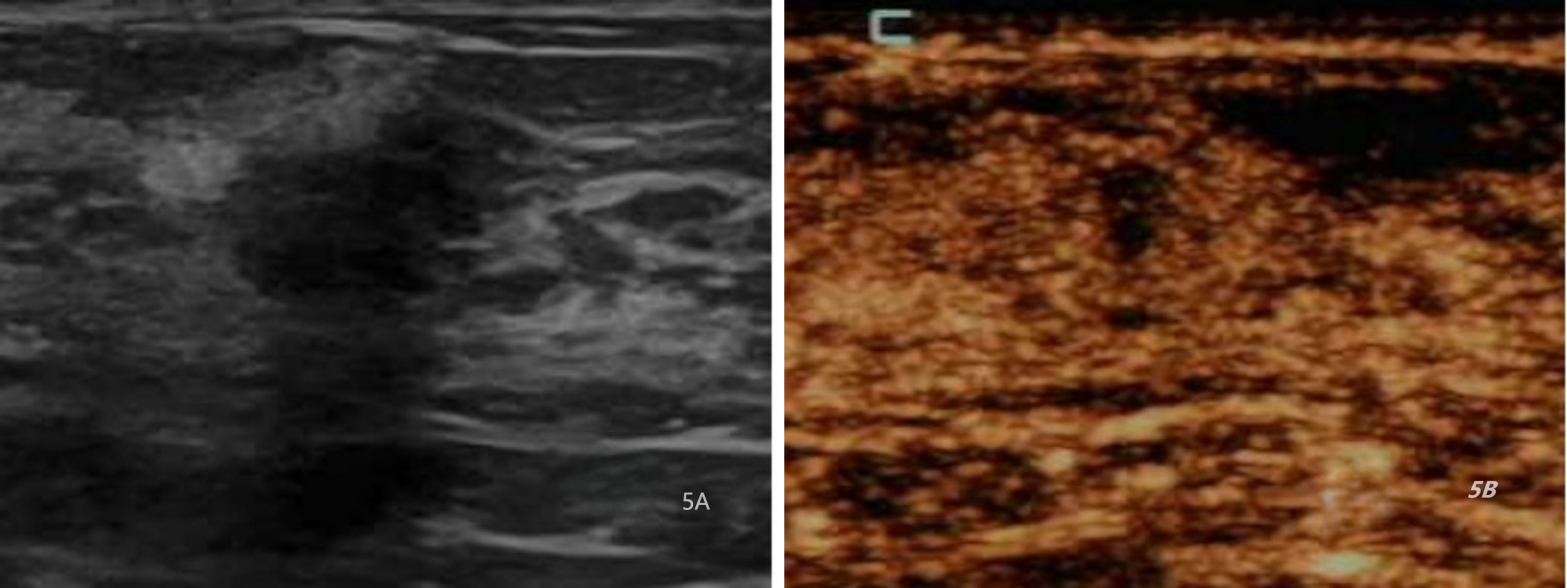
Ultrasound and CEUS images of a 29-year-old female patient with adenosis. **(A)**. Ultrasound (US) image indicating a hypoechoic lesion with unclear borders and irregular morphology., resulting in a total score of 8. **(B)** Contrast-enhanced ultrasound (CEUS) image depicting homogeneous equal renhancement, indistinct enhancement borders, and the lesion develops a focal perfusion defect, resulting in a total score of 6.

**Figure 6:**
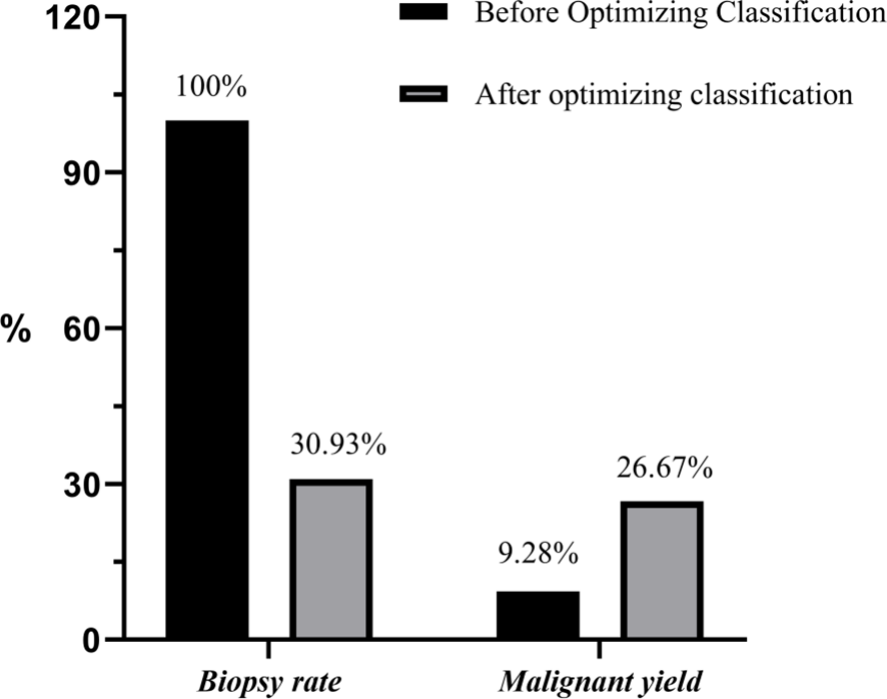
Comparison of biopsy rates and malignancy detection rates before and after optimized classification.

A total of 97 BI-RADS Category 4a lesions were analyzed, with 67 (69.07%) reclassified as Category 3 (**Figures 3**, **4**) and 30 (30.93%) remaining in Category 4a (**Figure 5**) following the optimized classification. This means that among 100 patients with category 4a lesions, nearly 70 could avoid unnecessary biopsy. The malignancy rate for Category 4a nodules increased from 9.28% before optimization to 26.67% after optimization. These findings indicate that the development of a scoring model can effectively identify high-risk patients and quickly and accurately detect malignant cases among 4a nodules, thereby assisting clinical decision-making. Moreover, the risk of malignancy in breast lesions reclassified as BI-RADS category three does not exceed 2%(1/67). Detailed results are presented in **Table 6** and **Figure 6**.

**Table 6:** Distribution of pathology types for 97 BI-RADS category 4a nodules after optimized classification.

Methods	Gauge	Pathological type n (%)	
		Benign	Malignant
Optimization of classification criteria	Below the cutoff value for both (Category 3)	66 (98.51)	1 (1.49)
	Above at least one cutoff value (Category 4a)	22 (73.33)	8 (26.67)

Data are the number of lesions with percentages in parentheses.

### 3.4 Case disclosure of missed tumour diagnoses and clinical outcome analysis

When reclassifying BI-RADS 4a nodules using a scoring system, one malignant nodule was reclassified as category three for follow-up, resulting in a false-negative rate of 1.49%. One year later, the patient underwent breast nodule excision, with pathology confirming a low-grade invasive early-stage Mucous carcinoma. Postoperative recovery was favourable. **Table 7** details the pathological type, imaging characteristics, CEUS score, clinical management, and follow-up outcome for the single case of a missed malignant nodule.

**Table 7:** Case-by-case disclosure of missed tumour diagnoses.

Case	Age (year)	Histological type	Nodule size (mm)	Contrast-enhanced ultrasound findings (score)	Conventional ultrasound findings (score)
1	54	Mucous carcinoma	11 × 12	Clear margins (1 point), regular shape (1 point), homogeneous echotexture (1 point), RI < 0.7 (0 points), no hyperechoic halo (0 points)	Low enhancement (1 point), clear post-enhancement margins (1 point), presence of perfusion defects (1 point), no enlargement of the lesion after enhancement (2 points), absence of crab-claw sign (0 points)

## 4 Discussion

Hypoxia plays a critical role in tumorigenesis. Previous studies have indicated that high-altitude regions, characterized by cold temperatures, low oxygen levels, high ultraviolet radiation, and dietary patterns rich in fat and low in fiber, can influence gene expression and increase cancer risk (3, 21–23). Although the BI-RADS has proven clinical utility in managing breast disease, the malignancy detection rate for BI-RADS Category 4a nodules, for which puncture biopsy is recommended, remains below 10%. In high-altitude areas, unique challenges such as information barriers, low literacy levels, limited access to medical knowledge, and the coexistence of Chinese, Western, and Tibetan medical practices complicate adherence to established guidelines and reduce compliance with recommended puncture biopsies. These factors highlight the need for innovative approaches to minimize unnecessary biopsies while ensuring accurate diagnosis. Optimizing the classification of BI-RADS 4a nodules could address this issue by improving diagnostic precision and reducing patient burden in resource-limited settings.

In this study, we observed that breast cancer in high-altitude regions predominantly exhibited US features such as unclear borders (73/127), irregular morphology (90/127), very hypoechoic areas, or glandular structural disorder (104/127), RI ≥ 0.7 (76/127), and peripheral hypoechoic halo (41/127). On CEUS, common findings included hyper-enhancement (96/127), unclear borders post-enhancement (80/127), enlargement post-enhancement (95/127), perfusion defects (44/127), and the presence of a crab-foot sign (69/127). These findings differ from those reported in previous studies (20, 24, 25), suggesting that the unique environmental factors of high-altitude regions may influence breast cancer imaging characteristics. The observed US characteristics may be attributed to the lack of an external envelope in breast cancer, where cancer cells infiltrate and grow chaotically along glandular lobes, ducts, and surrounding mesenchyme. This disorganized growth manifests ultrasonographically as unclear borders, irregular morphology, and the crab-foot sign. Hypoxia is a significant factor in tumor pathogenesis. Under hypoxic conditions, the body induces vascular endothelial growth factor expression, promoting neovascularization. These newly formed vessels are brittle, poorly elastic, and prone to leakage (26). Additionally, the unique high-fat, high-protein, and low-fiber diet of plateau region inhabitants (27) contributes to obesity, which reprograms neutrophils and disrupts vascular endothelial adhesion. This increases vascular wall permeability, exacerbating intratumoral hypoxia (28). The specific hypoxic environment in high-altitude regions facilitates excessive neovascularization in breast cancer tissue, which appears as hyper-enhancement on ultrasonography. Structurally abnormal neovascularization often results in numerous arteriovenous shunts and cancer embolisms, impeding venous return. This leads to uneven tumor microcirculation and increased diastolic blood flow resistance, causing perfusion defects visible on CEUS. Literature reports (29) suggest that hypoxic breast cancer cells produce a large number of heterogeneous, poorly differentiated cells. The resulting tissue exhibits significantly lower density compared to surrounding normal tissues, making the lesion area appear hypoechoic or very hypoechoic on US. Obesity further exacerbates cell proliferation, invasion, and inflammatory activity in cancer cells (30, 31), creating a more complex tumor microenvironment with increased inflammatory cell components such as neutrophils, mast cells, and lymphocytes. This inflammatory response often produces a strong peritumoral reaction, visible as a hyperechoic halo on US. Moreover, breast cancer stimulates the secretion of endothelial growth factor, promoting extensive neovascularization, particularly at the tumor periphery. Compared to conventional US, CEUS more accurately measures tumor diameter, as neovascularization in the peripheral region often appears enhanced, resulting in an enlarged enhancement range (32, 33).

The results of this study indicated no significant differences in the identification of benign and malignant breast nodules regarding echo attenuation, aspect ratio > 1, microcalcification, enhancement temporal phase, and enhancement homogeneity. This suggests a certain degree of overlap in these US features between benign and malignant lesions. Breast cancer tissue in high-altitude areas is often more vascularized and prone to hemorrhage, necrosis, or cystic degeneration (22). These characteristics, combined with a softer tissue texture, may reduce the likelihood of posterior echo attenuation in malignant lesions. Besides, a significant proportion of nodules in this study were adenopathic (36/148), which have a complex pathogenesis involving active connective tissue hyperplasia, lobular fibrosis, or localized calcification. Such features can sometimes mimic malignancy and present with posterior echo attenuation. Proliferative lesions (54/148) and inflammatory lesions (22/148) also accounted for a large proportion of benign nodules. These lesions often exhibit inhomogeneous and highly enhanced malignant-like features. Factors such as different stages of inflammation, varying extents of internal microabscesses, and ductal dilatation in adenopathy can affect enhancement homogeneity. Additionally, the cold climate of high-altitude regions may cause vascular contraction, resulting in thinner, more fragile neovascularization and slower blood flow within tumors. These factors could lead to benign-like features, such as iso-enhancement or low enhancement, in breast cancer nodules. The medical conditions in highland areas are limited, and nodules are often larger. An aspect ratio > 1 is usually associated with small cancerous foci originating in terminal ductal lobular units (< 1 cm in diameter). As tumors grow beyond 1 cm in diameter, the aspect ratio > 1 sign tends to disappear (34). In this study, the majority of malignant nodules (87/127) exceeded 1 cm in diameter. Microcalcifications are primarily formed due to local tumor dystrophy, necrosis, and cell lysis. The breakdown of nucleic acids in necrotic areas increases phosphate production, which, combined with elevated local calcium ion concentrations, results in calcium phosphate deposition. Lu Yang et al. (6, 35–37) observed that punctate calcifications can originate from both benign lesions and secretions within malignant luminal structures. Consequently, the role of microcalcifications in distinguishing between benign and malignant breast lesions requires further investigation.

Although conventional US and CEUS are valuable tools for differentiating between benign and malignant breast nodules, their interpretation remains highly subjective, heavily influenced by the physician’s experience and the quality of the instrumentation. These challenges are further compounded by the limited medical resources in high-altitude regions, increasing the risk of misclassification. In this study, quantitative scoring of US features improved diagnostic precision. The AUC for conventional US and CEUS were 0.963 and 0.839, respectively, with an optimal cutoff value of 7.5 points. These findings suggest that quantitative scoring can serve as a reliable index for optimizing the classification of breast nodules, thereby reducing the number of unnecessary biopsies.

This study found that conventional ultrasound demonstrated superior diagnostic efficacy compared with contrast-enhanced ultrasound, making it the preferred initial screening modality for BI-RADS 4a nodules in high-altitude regions. Notably, both examination methods yielded identical optimal cutoff values (7.5 points), significantly simplifying the clinical decision-making process. When either score reaches ≥ 7.5 points, indicating an elevated risk of malignancy, prioritization of needle biopsy is recommended. Where both scores are < 7.5, the nodule may be downgraded to BI-RADS category three, with follow-up recommended at 3–6 month intervals. In clinical practice, conventional ultrasound scoring may be prioritized for decision-making. Its high sensitivity of 93.7% ensures effective detection of most malignant nodules, while its specificity of 87.2% substantially reduces unnecessary biopsies. For complex nodules in which conventional ultrasound cannot clarify characteristics, the microcirculatory hemodynamic information provided by contrast-enhanced ultrasound serves as an effective differential diagnostic tool. However, one mucinous carcinoma was misclassified as Category 3. Mucinous carcinomas often exhibit round or oval shapes with clear borders, mimicking benign characteristics. These features can make them less likely to draw clinical attention, particularly in older women with loose breast tissue, and should be carefully considered as a potential diagnosis (33).

This study has several limitations. First, this study is a single-center retrospective investigation, with all samples originating from the same medical institution. Thereby introducing selection bias. Second, there is an imbalance in the disease spectrum, with invasive ductal carcinoma representing the majority of malignant lesions (80.5%), fibroadenomas (30.5%), and hyperplastic lesions (36.5%) predominating among benign lesions. In addition, the presence of benign lesions (such as adenosis and hyperplastic lesions) that demonstrate overlapping ultrasound features with malignant lesions may introduce bias into the scoring model when distinguishing specific benign subtypes. This may consequently compromise the stability of specificity. Therefore, the clinical translation of the ultrasound scoring model developed in this study may be significantly affected. Despite these limitations, to validate the model’s generalizability, we shall undertake multi-dimensional external validation (1): Incorporating medical centers across different altitude gradients (such as mid-altitude and low-altitude regions) to investigate whether altitude influences the interpretation thresholds of ultrasound features; (2) testing the model using ultrasound equipment of different brands and models to assess its stability across diverse imaging platforms; (3) conducting prospective workflow trials to validate the operational feasibility and diagnostic consistency of the scoring system in real-world clinical settings.We recognize that successful clinical translation of a scoring model requires the establishment of standardized operational procedures. The first step is to develop training protocols for sonographers to ensure a consistent understanding of BI-RADS classification criteria and the definitions of features included in this scoring system. The second step involves quantifying inter-reader variability among physicians of differing levels of experience through regular consistency assessments (for example, Kappa coefficient evaluations). The third step is to implement a monthly or quarterly audit, including random sampling of scoring records and cross-referencing with pathological outcomes, thereby establishing a continuous quality control feedback loop. Only through such systematic validation and standardized implementation can this scoring model progress from statistical reporting to routine clinical practice.

## Data Availability

The raw data supporting the conclusions of this article will be made available by the authors, without undue reservation.
